# Supplementary material for: HIVprotI: an integrated web based platform for prediction and design of HIV proteins inhibitors
Source: J Cheminform. 2018 Mar 9;10:12. doi: 10.1186/s13321-018-0266-y (PMC5845081; doi:10.1186/s13321-018-0266-y)
Supplement: Supplementary file 2 — Additional file 2. Source code of HIVProtI web server. [file 13321_2018_266_MOESM2_ESM.zip › HIVprotI_Source-code/hivprotI/weka-3-6-6/doc/weka/gui/graphvisualizer/GraphNode.html]

GraphNode


---


|  |  |  |
| --- | --- | --- |
| **Overview** \| **Package** \| **Class** \| **Tree** \| **Deprecated** \| **Index** \| **Help** \| **Weka's home** | |  |
| **PREV CLASS**   **NEXT CLASS** | **FRAMES**    **NO FRAMES**     **All Classes** |  |
| SUMMARY: NESTED \| FIELD \| CONSTR \| METHOD | DETAIL: FIELD \| CONSTR \| METHOD |  |


---


## weka.gui.graphvisualizer Class GraphNode

```
java.lang.Object
  weka.gui.graphvisualizer.GraphNode
```

**All Implemented Interfaces:**: GraphConstants

---

``` public class GraphNode extends java.lang.Object implements GraphConstants ```

This class represents a node in the Graph.

**Version:**
:   $Revision: 1.5 $ - 23 Apr 2003 - Initial version (Ashraf M. Kibriya)

**Author:**
:   Ashraf M. Kibriya (amk14@cs.waikato.ac.nz)

---

| **Field Summary** | |
| --- | --- |
| `int` | `nodeType`             Type of node. |
| `int` | `x`             The x and y position of the node |
| `int` | `y`             The x and y position of the node |

**Fields inherited from interface weka.gui.graphvisualizer.GraphConstants**

`DIRECTED, DOUBLE, NORMAL, PLURAL_DUMMY, REVERSED, SINGULAR_DUMMY`


**Constructor Summary**

`GraphNode(java.lang.String id,
java.lang.String label)`
  
          Constructor

`GraphNode(java.lang.String id,
java.lang.String label,
int type)`
  
          Constructor


| **Method Summary** | |
| --- | --- |
| `boolean` | `equals(java.lang.Object n)`             Returns true if passed in argument is an instance of GraphNode and is equal to this node. |

**Methods inherited from class java.lang.Object**

`getClass, hashCode, notify, notifyAll, toString, wait, wait, wait`

**Field Detail**

### x

```
public int x
```

:   The x and y position of the node

---


### y

```
public int y
```

:   The x and y position of the node

---


### nodeType

```
public int nodeType
```

:   Type of node. Default is Normal node type


**Constructor Detail**

### GraphNode

```
public GraphNode(java.lang.String id,
                 java.lang.String label)
```

:   Constructor

---


### GraphNode

```
public GraphNode(java.lang.String id,
                 java.lang.String label,
                 int type)
```

:   Constructor


**Method Detail**

### equals

```
public boolean equals(java.lang.Object n)
```

:   Returns true if passed in argument is an instance
    of GraphNode and is equal to this node.
    Implemented to enable the use of contains method
    in Vector/FastVector class.

    :   **Overrides:**: `equals` in class `java.lang.Object`


---


|  |  |  |
| --- | --- | --- |
| **Overview** \| **Package** \| **Class** \| **Tree** \| **Deprecated** \| **Index** \| **Help** \| **Weka's home** | |  |
| **PREV CLASS**   **NEXT CLASS** | **FRAMES**    **NO FRAMES**     **All Classes** |  |
| SUMMARY: NESTED \| FIELD \| CONSTR \| METHOD | DETAIL: FIELD \| CONSTR \| METHOD |  |


---
